# A systematic review and meta-analysis of the effects of long-term antibiotic use on cognitive outcomes

**DOI:** 10.1038/s41598-024-54553-4

**Published:** 2024-02-18

**Authors:** Yongqin Ye, Hor Yee Kimberley Tong, Wai Hong Chong, Zhiqian Li, Paul Kwong Hang Tam, Daniel T. Baptista-Hon, Olivia Monteiro

**Affiliations:** 1 Faculty of Medicine, Medical Sciences Division, Macau University of Science and Technology, Avenida da Harmonia, Praia Park, Coloane, 999078 Macao SAR China; 2 Faculty of Medicine, The Chinese University of Hong Kong, Shatin, Hong Kong SAR China; 3 Division of Systems Medicine, School of Medicine, University of Dundee, Dundee, UK

**Keywords:** Neuroscience, Medical research

## Abstract

Antibiotics are indispensable to infection management. However, use of antibiotics can cause gut microbiota dysbiosis, which has been linked to cognitive impairment by disrupting communication between the gut microbiota and the brain. We conducted a systematic review and meta-analysis on the effects of long-term antibiotic use on cognitive outcomes. We have searched PubMed, Web of Science, Embase, Cochrane Library and Scopus for English publications before March 2023 following the PRISMA guidelines. Screening, data extraction, and quality assessment were performed in duplicate. 960 articles were screened and 16 studies which evaluated the effect of any antibiotic compared to no antibiotics or placebo were included. Case-reports, in vitro and animal studies were excluded. We found that antibiotic use was associated with worse cognitive outcomes with a pooled effect estimate of − 0.11 (95% CI − 0.15, − 0.07, Z = 5.45; P < 0.00001). Subgroup analyses performed on adult vs pediatric patients showed a similar association of antibiotic on cognition in both subgroups. Antibiotic treatment was not associated with worse cognition on subjects with existing cognitive impairment. On the other hand, antibiotic treatment on subjects with no prior cognitive impairment was associated with worse cognitive performance later in life. This calls for future well-designed and well-powered studies to investigate the impact of antibiotics on cognitive performance.

## Introduction

Dementia can occur in people of all ages and is a leading cause of death and disability worldwide accounting for > 1.6 million total deaths in 2019 (*WHO Global Health Estimates*). It is a chronic and progressive deterioration in cognitive function beyond that to be expected from normal biological aging. Whilst dementia occurs mainly at older ages, it is not an inevitable consequence of ageing^1^. Dementia is usually a result of neurodegenerative and neurological diseases such as Alzheimer’s disease (AD), Parkinson’s disease or stroke. Death or degeneration of neurons in brain areas important for memory and cognition is the main cause for loss of cognitive function. AD accounts for around 70% of all cases of dementia worldwide. Although extensive research and progress has been made regarding the pathophysiology of AD, its cause is not fully understood. There is currently no cure for AD or dementia and treatment focuses on the alleviation of symptoms and risk reduction.

There is growing interest on the regulation of the gut-brain axis and how dysbiosis in the intestines can affect brain function. This raises the possibility that the microbiota–gut–brain-axis has a role to play in neurodegenerative diseases^2^. In addition to the important role of the gut microbiota on metabolic functions^3^, the gut microbiota is capable of synthesizing and releasing neurotransmitters (e.g. GABA and tryptophan) and neuromodulators such as short-chain fatty acids and biogenic amines (e.g. serotonin, histamine, and dopamine)^4^. Moreover, the gut microbiota produces proinflammatory cytokines that can activate neuroinflammation which may affect cognitive function^5^. The relative composition of the gut microbiota is affected by our genetic makeup, lifestyle, diet, and drugs we take such as antibiotics^6^. There is also evidence that the composition of gut microbiota changes with aging^7^, and the microbiota composition in children may be less stable and diverse compared to adults^8^. It is therefore possible that perturbations in microbiota compositions and their effects may be age dependent. There are profound changes in the gut microbial profiles in AD patients compared to healthy controls with less richness in operational taxonomic units in AD and less α- and β-diversity (mean diversity of species and ratio between regional and local species)^9^. There is increasing evidence that cognitive impairments caused by antibiotics are effects of disruption of the gut microbiota. Germ-free mice with no intestinal microbiome displayed disrupted brain development^10^, suggesting a direct link between the microbiome and the brain. There is strong evidence that long-term treatment with broad spectrum antibiotics disrupted the microbial composition in the gut^11^. The loss of diversity in gut microbiota and the altered microbial composition after long-term antibiotic treatment may be long lasting, where bacterial populations do not recover after cessation of antibiotics^12^. A 2-week treatment of rheumatoid arthritis patients with vancomycin showed an inability of some patients to fully recover their baseline microbiota structure up to 22 weeks after antibiotic cessation^13^. Importantly, development of AD seems to be directly linked to the gut microbiome. Transplantation of healthy fecal microbiota to AD mice reduced brain deposition of amyloid-beta, decreased tau phosphorylation, increased synaptic plasticity and improved cognitive performance^14^. In a clinical case report, an AD patient with mild cognitive impairment who received a single fecal microbiota transplantation (FMT) had an increased mental acuity and improved affection 2 months after FMT^15^. Furthermore, memory and mood were improved at 4 and 6 months after FMT with improvements in MMSE scores compared to scores prior to FMT. Another recent case report of the use of FMT in a patient with AD dementia confirmed this finding^16^. As rapidly as 1 month after FMT, the patient benefited from improved cognitive functions including improvements in short-term memory, semantic skills, attention, non-verbal learning, and expressive affection.

Antibiotics play a crucial role in modern medicine to prevent serious complications and fatality from infections. The average total antibacterial consumption in the European Union (EU) for 2019 was 19.4 daily doses per 1000 inhabitants per day (Ecdc. Antimicrobial consumption in the EU and EEA). In the United States, 270.2 million antibiotic prescriptions were written in 2016, a rate which was equivalent to enough antibiotic courses for 5 out of every 6 Americans (U.S. Department of Health and Human Services, 2018). More than 10% of children in the EU use antibiotics each year and antibiotics account for 25% of all pediatric prescriptions in the United States^17^. Although antibiotics are indispensable to control infections, its use can cause gut microbiota dysbiosis and the potential downstream cognitive impairments^18^. This may be especially true for broad-spectrum antibiotics^19^. Animal studies found that antibiotic treatment impaired cognition by disrupting communication between the gut microbiota and the brain^20^. Recently, several human studies also found that antibiotic use reduced cognitive function later in life^18,21–23^. On the other hand, there are clinical trials investigating the efficacy of antibiotics in the treatment of dementia (NCT03413384, NCT04408625, NCT04629495, NCT04200911). Broad spectrum antibiotics such as doxycycline, rifampin and minocycline have been tested for their ability to reduce cognitive decline in patients with neurodegenerative diseases^24–28^. In vitro studies suggest rifampin and its derivative prevented the aggregation of amyloid beta peptide, prevented beta-amyloid fibrils formation and reduced neurotoxic effects of amyloid beta by acting as a free radical scavenger^29^. In vivo animal studies also supported the role of rifampin and its derivative in clearing amyloid-beta and tau oligomers and improved spatial memory in AD mouse models^30^. Doxycycline disrupts the formation of amyloid-beta plaques in AD models by destabilising the structure of amyloid fibrils and reducing neuroinflammation leading to rescue of memory impairments in AD mice^31^. Minocycline, another tetracycline antibiotic, prevented neurotoxicity in AD mouse models by reducing deposition and fibrillisation of amyloid-beta, reducing tau aggregation and reducing inflammatory markers in AD brains leading to rescue of memory impairments^32^.

Antibiotic stewardship for better antibiotic prescription is an important component of infection management, primarily to mitigate the emerging problem of antibiotic resistance. However, the possibility of cognitive impairment with long-term and recurrent antibiotic use, if present, will also be an important consideration in antibiotic stewardship. We therefore initiate this systematic review with the aim of evaluating the current clinical evidence on antibiotic usage and cognitive outcomes in patients who have received long-term or recurrent antibiotic treatment.

## Results

**Figure 1:**
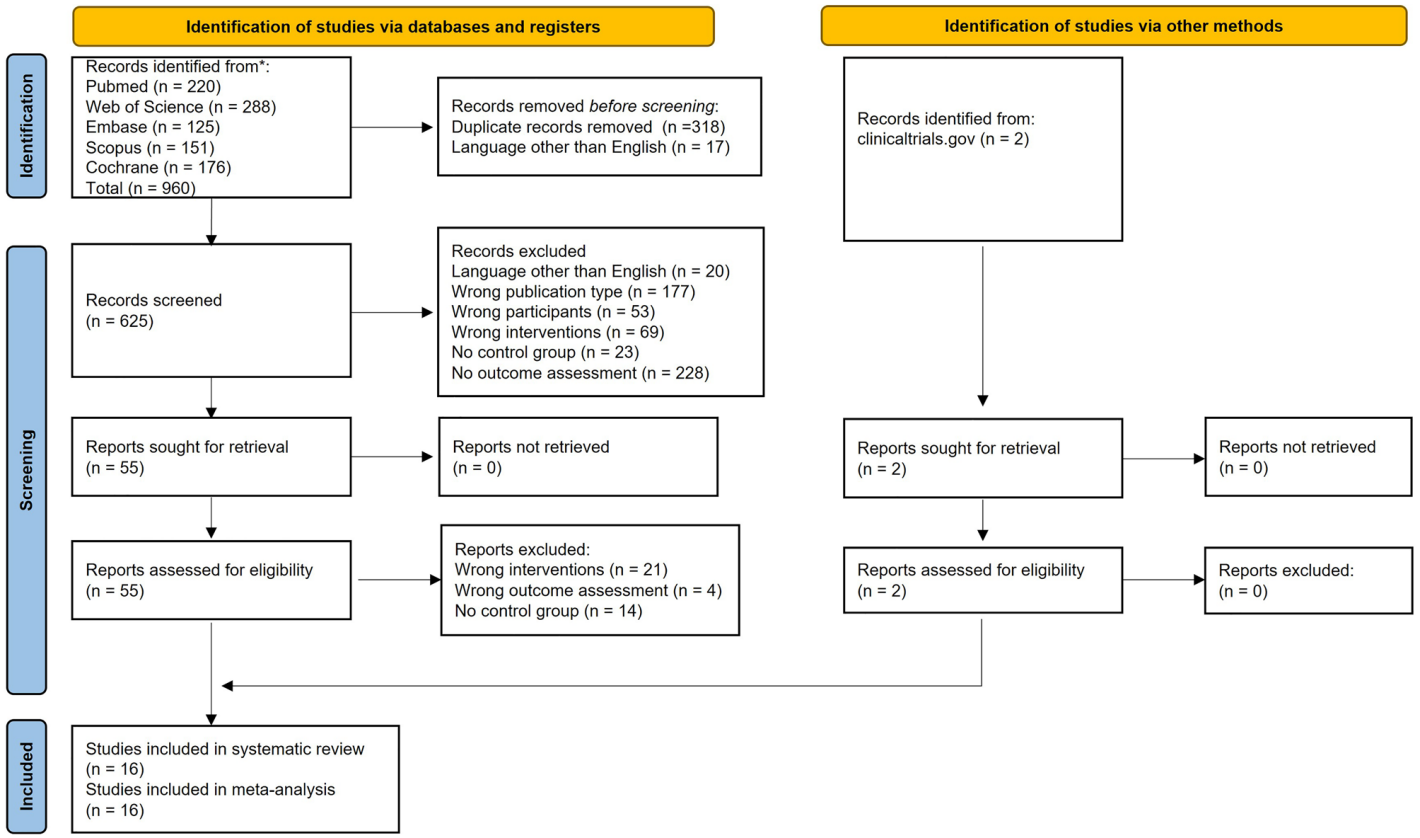
PRISMA flowchart for study selection according to Preferred Reporting Items for Systematic Reviews and Meta-Analyses (PRISMA) 2020.

We identified a total of 960 articles (220 from Pubmed, 288 from Web of Science, 125 from Embase, 151 from Scopus and 176 from the Cochrane Library) from electronic sources for this systematic review (flowchart is shown in Fig. 1).

We found two peer reviewed publications from two additional articles identified from records on http://www.clinicaltrials.gov. After removal of duplicates and removal of non-English articles, title and abstract screening was performed for 625 articles. A preliminary screen was carried out to assess for eligibility. 55 articles were selected for full text screening, after 399 articles were removed due to wrong intervention, no assessment outcome or no control. Finally, after removal of articles with the wrong intervention (including articles that did not specify the type of antibiotics used or the duration or frequency of antibiotic use, or the intervention was not an antibiotic), outcome assessment (including articles where we could not identify the treatment group from the outcome assessment), and articles with no control group, 16 articles were included in the systematic review and meta-analysis^18,21–27,33–39^. Descriptive summaries of the 16 included studies are listed in Table 1. For all randomised controlled trials, one or two specific antibiotic was tested. Minocycline, doxycycline and rifampin were the most common antibiotics tested^24–28,39^. Five retrospective cohort studies investigated long-term antibiotic use (< 7 to 28 days^38^, 30 to ≥ 91 days^33^, < 7 to ≥ 15 days^36^, and < 30 to ≥ 365 days^37^) and the risk of developing dementia later in life. Our primary objective is in understanding the long-term effects of antibiotic treatment on cognitive performance. Hence, where studies reported more than one duration of antibiotic treatment, the longest duration was analysed (Table 1). By extracting data on the longest treatment duration, we aim to capture the potential cumulative and sustained impact of antibiotics on cognitive function. Where studies reported more than one specified antibiotic treatment, the effects of each antibiotic was analysed. Two additional cohort studies^18,23^ investigated long-term or recurrent antibiotic use for > 2 months or 3 regimens in 1 year with no specification of the types of antibiotics used. Three studies investigated the effects of paediatric long-term or recurrent antibiotic use^21–23^. The included studies also showed differences in their interval to testing, which represents the time between the commencement of antibiotic treatment and cognitive tests carried out. The timing of the cognitive tests can be divided into acute (cognitive performance was measured at the end of long-term antibiotic treatment) or chronic (cognitive tests performed at least 6 months after treatment).

**Table 1:** Descriptive summary of included studies.

	Study	Type of study	Abx used	Dosage of Abx used	DOT	Sample size		Mean age (years)	Interval to testing	Underlying cognitive-related deficits	Outcome	Acute/chronic effect	Risk of Bias	SMD *Ln RR*
						Abx	Ctrl							
1a	Chao et al. 2022^38^	RCS	Beta-lactam	Var	> 28 days	31	36	63.4	1–16 years	None	Dementia diagnosis according to the DSMMS	Chronic	Low	*− 0.879*
1b	Chao et al. 2022^38^	RCS	Ceph	Var	> 28 days	36	36	63.4	1–16 years	None	Dementia diagnosis according to the DSMMS	Chronic	Low	*− 0.816*
1c	Chao et al. 2022^38^	RCS	Dox	Var	> 28 days	31	36	63.4	1–16 years	None	Dementia diagnosis according to the DSMMS	Chronic	Low	*− 0.865*
2	Howard et al. 2020^24^	RCT	Min	400 mg daily	24 months	119	140	74.3	24 months	Mild AD***	sMMSE	Acute	Mod	0.168
3	Kelly et al. 2015^39^	RCT	Min	50 mg BID 1st week; 100 mg BID after	10 weeks	29	23	42.9	10 weeks	Schizophrenia	MCCB	Acute	Low	*− *0.698
4	Kim et al. 2022^33^	RCS	NS	NS	≥ 91 days	124	75,619	57.5	10 years	None	Dementia diagnosis according to ICD-10^#^	Chronic	Mod	*− 0.207*
5	Kraig et al. 2018^34^	RCT	Rapa	1 mg daily	8 weeks	12	14	80.4	16 weeks	None	SLUMS	Acute	Low	*− *0.096
6	Liu et al. 2022^23^	RCS	NS	NS	NS^†^	5026	30,859	3–19 years^†^	> 18 years	None	FI	Chronic	Mod	*− *0.091
7	Loeb et al. 2004^25^	RCT	Dox (D) and Rif (R)	200 mg (D) + 300 mg (R) daily	12 months	43	39	76	12 months	Mild to moderate AD**	SADAS-cog	Chronic	Low	0.396
8	Mataix-Cols et al. 2014^35^	RCT	d-Cyclo-serine	50 mg 10 times in 17 weeks	12 months	13	14	14.7	12 months	Pediatric OCD	CY-BOCS	Chronic	Low	*− *0.196
9	Mehta et al. 2022^18^	PCS	NS	NS	> 2 months	1195	3398	54.7	7 years	None	CogState	Chronic	Mod	*− *0.117
10a	Molloy et al. 2013^26^	RCT	Dox	100 mg BID	12 months	102	102	≥ 50	12 months	Mild to moderate AD*	SADAS-cog	Acute	High	*− *0.468
10b	Molloy et al. 2013^26^	RCT	Rif	300 mg daily	12 months	101	102	≥ 50	12 months	Mild to moderate AD*	SADAS-cog	Acute	High	*− *0.469
11a	Ou et al. 2021^36^	RCS	Mac	Var	≥ 15 days	1657^♐^	4971^♐^	65.5	≥ 1 year	None	Dementia diagnosis according to the ICD-9-CM	Chronic	Low	*− 0.181*
11b	Ou et al. 2021^36^	RCS	FQ	Var	≥ 15 days	1657^♐^	4971^♐^	65.5	≥ 1 year	None	Dementia diagnosis according to the ICD-9-CM	Chronic	Low	*− 0.264*
12	Ruiz-Antoran et al. 2018^27^	RCT	Min	3 mg/kg/d	24 weeks	10	11	12	24 weeks	Angelman syndrome	DI-MPR	Acute	Low	*− *0.832
13	Sacktor et al. 2011^28^	RCT	Min	100 mg orally/12 h	24 weeks	52	55	51	24 weeks	HIV-associated cognitive impairment	NPZ-8	Acute	Mod	*− *0.072
14	Slykerman et al. 2017^21^	Case Ctrl	NS	NS	NS^†^	369	26	1	11 years	None	WISC	Chronic	Mod	*− *0.278
15	Slykerman et al. 2019^22^	PCS	NS	NS	NS d^†^	487	70	< 6 m	11 years	None	WISC	Chronic	Low	*− *0.264
16a	Yang et al. 2021^37^	RCS	Sf	NS	≥ 365 days	6	9	62.1	≤ 15 years	None	Dementia diagnosis according to the ICD-9-CM	Chronic	Low	*− 0.070*
16b	Yang et al. 2021^37^	RCS	Clin	NS	≥ 365 days	6	9	62.1	≤ 15 years	None	Dementia diagnosis according to the ICD-9-CM	Chronic	Low	*− 0.409*

When cognitive outcome was measured at several time points during the course of the intervention, the longest duration of antibiotic use is analysed. If more than one dose of antibiotic was administered, the outcome for the highest dose was evaluated. *Abx* antibiotic, *AD* Alzheimer’s disease, *BALDS* Bristol Activities of Daily Living Scale, *Ceph* Cephalosporin, *Clin* Clindamycin, *Ctrl* Control, *CY-BOCS* Children’s Yale–Brown Obsessive Compulsive Scale, *DI-MPR* Development Index of the Merrill-Palmer Revised Scale, *DOT* Duration Of Treatment, *Dox* Doxycycline, *DSMMS* Diagnostic and Statistical Manual of Mental Disorders, *FI* Fluid intelligence, *FQ* Fluoroquinolones, *ICD-9-CM* International Classification of Diseases, 9th Revision, Clinical Modification, *ICD-10* 10th Revision of the International Classification of Diseases, *Mac* Macrolides, *MCCB* MATRICS Consensus Cognitive Battery, *Min* Minocycline, *Mod* Moderate, *ND* No Difference, *NPZ-8* Neuropsychological Test Composite z score, *NS* Not Specified, *OCD* Obsessive Compulsive Disorder, *PCS* Prospective Cohort Study, *Rapa* Rapamycin, *RCS* Retrospective Cohort Study, *RCT* Randomised Controlled Trial, *Rif* Rifampin, *RR* Risk Ratio, *SADAScog* Standardized Alzheimer’s Disease Assessment Scale-Cognitive Subscale, *Sf* Sulfadiazine, *SLUMS* Saint Louis University Mental Status Exam, *SMD* Standardized Mean Difference, *sMMSE* standardized mini mental state examination, *Var* Various, *WISC* Wechsler Abbreviated Scale of Intelligence.

^#^Diagnosis of dementia according to ICD-10 codes of F00–F03 and G30 with prescription records of anticholinesterase drugs (memantine, donepezil, rivastigmine, or galantamine).

^†^Recurrent use of antibiotics as three or more separate prescriptions within a year.

^♐^Total number of participants in study.

* and **Mild to moderate AD was defined as sMMSE score 14–26 and 11–25.

***Mild AD defined as sMMSE score 26.4 ± 1.9 and BADLS score 5.6 ± 6.

Supplementary Fig. 1A summarises the risk of bias for 8 randomised controlled studies. Amongst the 8 RCT, one study Molloy et al*.*^26^ has a high risk of bias due to missing outcome data in the study. In addition, there are some concerns regarding selection of reported results Sacktor et al*.*^28^ and Howard et al*.*^24^. There were also some concerns regarding deviations from intended intervention and bias due to missing outcome data in Kraig et al*.*^34^. Among the seven cohort studies, five studies, Chao et al*.*^38^, Kim et al*.*^33^, Ou et al*.*^36^, Slykerman et al*.*^22^ and Yang et al*.* had low risk of bias. Liu et al*.*^23^ and Mehta et al*.*^18^ and, had a low to moderate risk of bias with scores of 6 and 7 respectively. Slykerman et al*.*^21^, a case control study, had a moderate risk of bias score of 5/9. Supplementary Fig. 1B summarises the risk of bias for all included studies.

**Figure 2:**
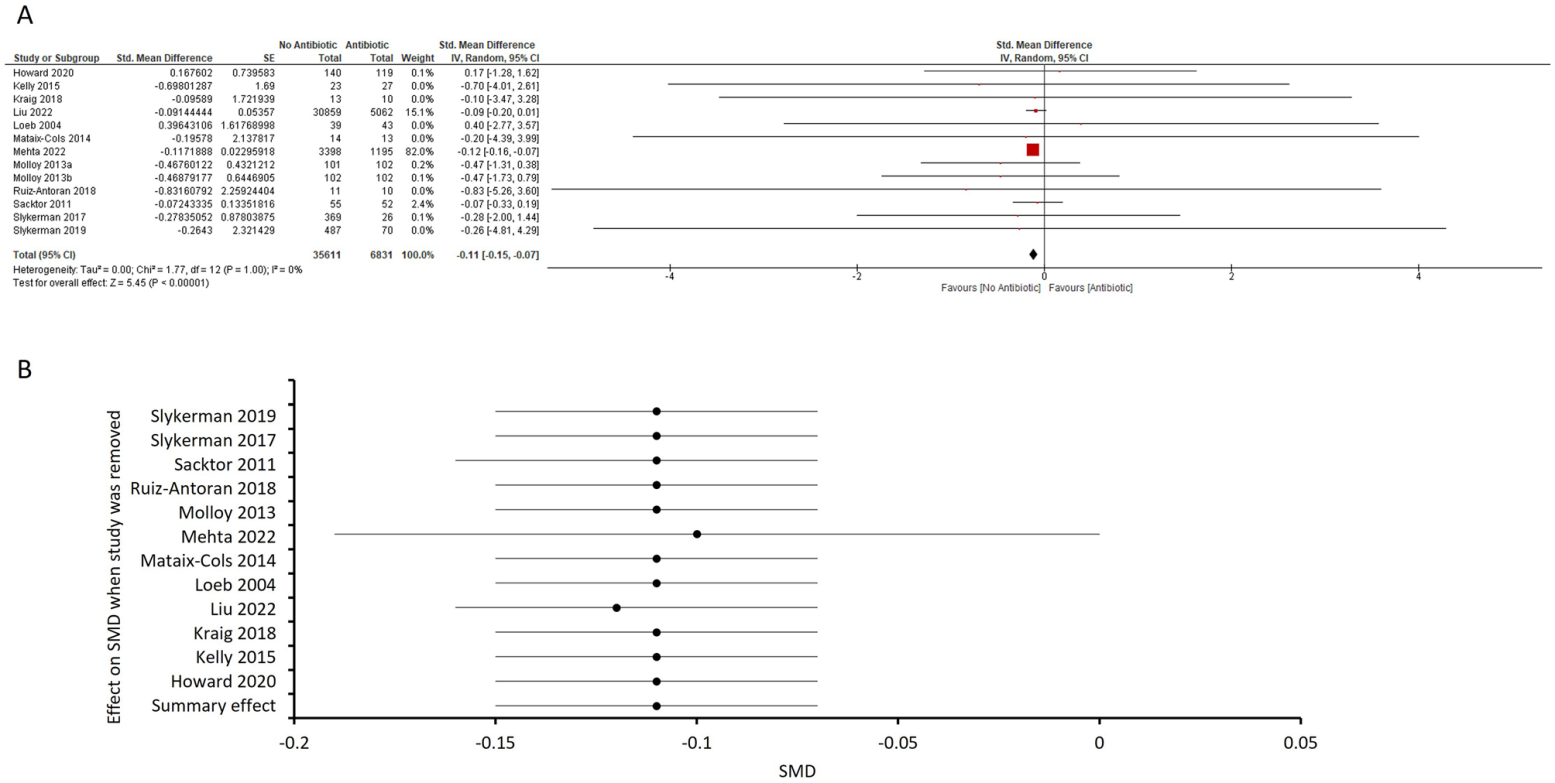
(**A**) Forest plot of adjusted SMDs showing overall effect of antibiotic on cognitive performance. One study (Molloy et al.^26^) tested two antibiotics on different populations and hence, the results were extracted separately. There is an overall effect favouring no antibiotic in the SMD of cognitive performance (pooled effect estimate − 0.11, 95% CI − 0.15, − 0.07; Z = 5.47; P < 0.00001). (**B**) Sensitivity analysis showing the change in SMD after the individual removal of each study from the overall analysis. Individual study was sequentially removed to evaluate their overall contribution to the pooled estimate or heterogeneity. Removing the study with the heaviest weighting (Mehta et al.^18^) revealed a smaller but nevertheless statistically significant overall effect (Z = 1.99; P = 0.05) of antibiotics on cognitive impairment. Removing the second largest study (Liu et al*.*^23^) altered the SMD in favour of no antibiotics (− 0.12, 95% CI − 0.16, − 0.07) with a similar overall effect (Z = 5.21; P < 0.00001). Removing other studies sequentially did not result in a change in the pooled SMD.

We included 16 studies in our meta-analysis, of which 8 were randomized controlled trials, 8 were cohort studies. We evaluated the standardized mean difference (SMD) of antibiotic exposure on cognitive test scores from 12 studies where the SMD can be calculated (Fig. 2A). One study, Molloy et al*.*, evaluated the effects of doxycycline and rifampin on cognition on separate cohorts and hence, data on the two antibiotics were analysed separately. The pooled effect estimate was − 0.11 [95% CI − 0.15, − 0.07, total number of control subjects = 35,611, antibiotic subjects = 6831]. A *Z*-test showed that antibiotic use was associated with worse cognitive outcomes (Z = 5.45; P < 0.00001; Fig. 2A). We found no heterogeneity in the included studies (τ^2^ = 0, χ^2^ = 1.77, P = 1, I^2^ = 0%). We did not perform publication bias analyses due to the small number of studies included. Visual inspection of the forest plot indicates that one large study carries most of the weight in the calculation of the pooled estimate. We therefore performed a sensitivity analysis (Fig. 2B), by sequentially removing individual studies to evaluate their overall contribution to the pooled estimate or heterogeneity. When we removed the study with the heaviest weighting, Mehta et al*.*^18^, the data revealed a smaller but nevertheless statistically significant overall effect (pooled effect estimate − 0.10, 95% CI − 0.19, 0.00; Z = 1.96; P = 0.05). We found minor effects of sequentially removing other studies on either the pooled SMD (from − 0.11 to − 0.12 when Liu et al*.*^23^ was removed), or extent of heterogeneity (I^2^ = 0% in all cases).

We separately analysed the effects of antibiotics on cognition for studies which reported hazard ratios for the likelihood of developing dementia after antibiotic treatment. Similarly, studies which evaluated the risk of dementia after more than one antibiotic treatment on separate cohorts were analysed separately. The log risk ratio for the four retrospective cohort studies were analysed using the generic inverse-variance method and the pooled effect estimate was 0.70 [95% CI 0.59, 0.83]. A *Z*-test showed that antibiotic use was again associated with worse cognitive outcomes (Z = 4.05; P < 0.0001; Supplementary Fig. 2A). Chi^2^ for heterogeneity revealed large differences in data (Chi^2^ = 138.33, P < 0.00001; I^2^ = 96%). This means that 96% of the variability in the effects observed is due to heterogeneity and not chance. Sensitivity analysis did not find a great deal of difference when studies were removed sequentially (risk ratios from 0.65 to 0.77; Supplementary Fig. 2B). When the study with the heaviest weight (Yang et al*.*^37^) was removed, the effect estimate was 0.65 [95% CI 0.58, 0.73] and the overall effect was Z = 7.36; P < 0.00001. Heterogeneity decreased slightly (Chi^2^ = 6.7, P < 0.00001; I^2^ = 25%). Yang et al*.* reported the risk of dementia is 0.932 and 0.664 [95% CI 0.901, 0.986; 0.602, 0.692] in patients who took sulfadiazine and those who took clindamycin for ≥ 365 days respectively. Unsurprisingly, removing this study resulted in a much lower I^2^ statistic.

**Figure 3:**
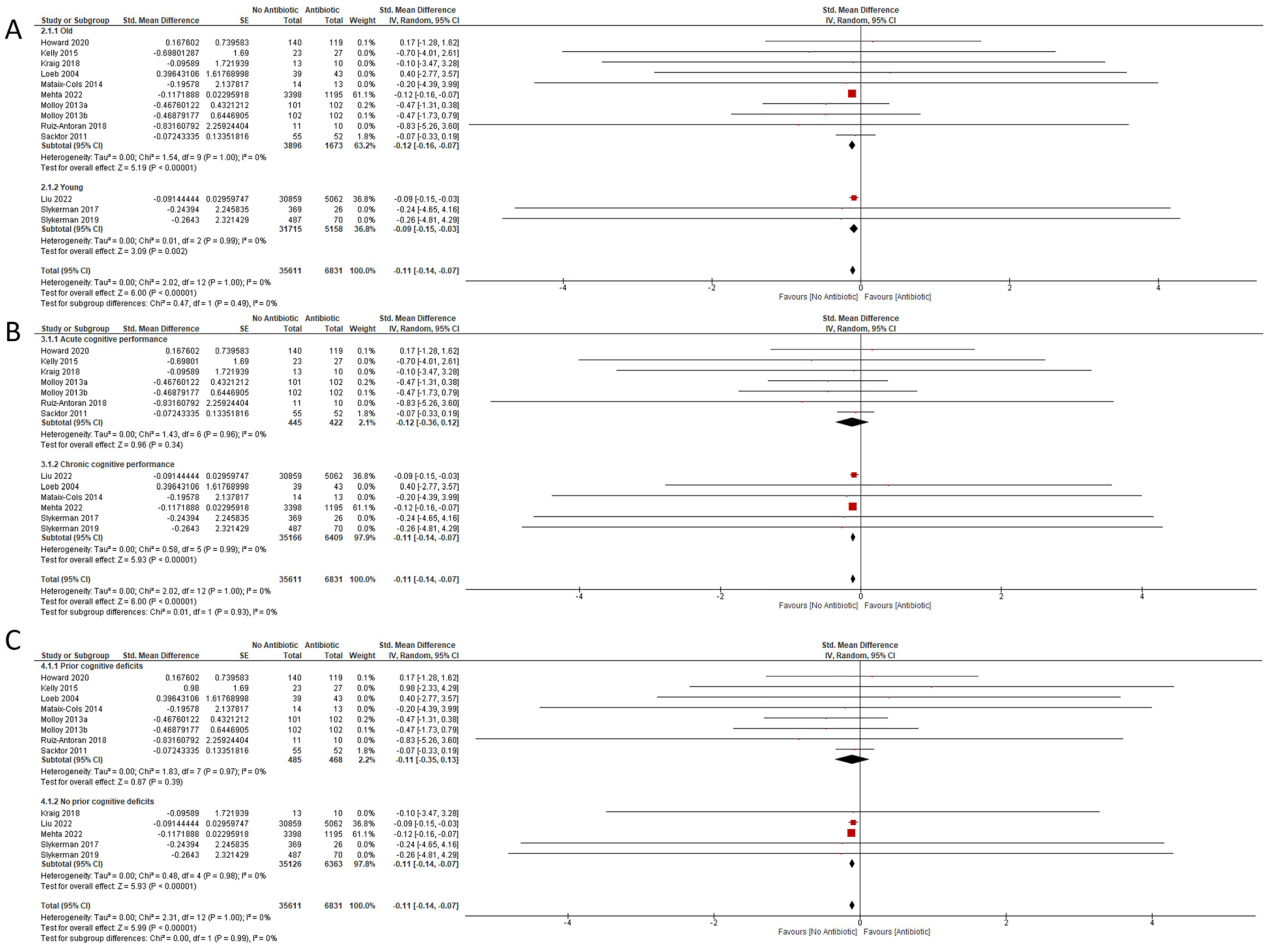
Forest plots of adjusted SMDs showing analysis (**A**) on antibiotic treatment in the pediatric and the adult subgroups. There is an overall effect favouring no antibiotic for the “Young” (Z = 3.09; P = 0.002) and the “Old” (Z = 5.20; P < 0.00001) subgroups. (**B**) Analysis on the acute and chronic effects of antibiotic treatment on cognitive performance. Cognitive performance was not affected (Z = 1.03; P = 0.31) when assessed immediately after antibiotic treatment. Cognitive performance was significantly impaired (Z = 5.93; P < 0.00001) when assessed > 6 months after antibiotic treatment. (**C**) Analysis on subgroups with and without prior cognitive deficits. Cognitive performance was not affected (Z = 0.94; P = 0.35) in the subgroup with prior cognitive deficits. Cognitive performance was significantly impaired (Z = 5.93; P < 0.00001) in the subgroup with no prior cognitive deficits.

The microbiota composition shows dynamic changes through life^22,23^. Children have less diverse gut microbial population which makes them more vulnerable to antibiotic dysbiosis^40^. Children may therefore be more vulnerable to the effects of antibiotics on cognitive abilities later in life. We carried out subgroup analysis for SMD in cognitive abilities for pediatric (under 19 years; 3 studies) and adults (10 studies). Studies reporting hazard ratios were not included in this subgroup analysis since there is no accurate way to convert hazard ratios to SMD. Antibiotic use had an overall effect on the adult subgroup (pooled effect estimate = − 0.12; 95% CI − 0.16, − 0.07; total number for control subjects = 3896, antibiotic subjects = 1673; Z = 5.19; P < 0.00001;) and the pediatric subgroup (pooled effect estimate = − 0.11; 95% CI − 0.14, − 0.07; total number for control subjects = 31,715, antibiotic subjects = 5158, Z = 3.09; P = 0.002) (Fig. 3A). There was no heterogeneity of data in both subgroups (I^2^ = 0%). The subgroup analysis indicated that long-term or recurrent antibiotic use had a negative influence on cognitive performance in children and adults.

The included studies contained cognitive performance measured soon after antibiotic treatment (7 studies), and also longer term (> 6 months) effects (6 studies). We therefore attempted to dissect acute and chronic effects of antibiotic treatment on cognitive performance. Studies reporting hazard ratios were not included in this subgroup analysis since there is no accurate way to compare hazard ratios to SMD. The acute effects group included studies in which cognitive tests were performed < 6 months after the end of antibiotic use. Table 1 details the interval between the first administration of antibiotics and testing of cognitive outcomes. The chronic effects group included studies in which cognitive tests were performed ≥ 6 months after the end of antibiotic use. The pooled SMD estimate for studies evaluating acute cognitive performance was − 0.12 [95% CI − 0.36, 0.12; total number for control subjects = 445, antibiotic subjects = 422], and was not statistically significant (Z = 0.96; P = 0.34; I^2^ = 0%; Fig. 3B). By contrast, the pooled SMD estimate for the studies evaluating chronic cognitive performance was − 0.11 [95% CI − 0.14, − 0.07; total number for control subjects = 35,166, antibiotic subjects = 6409], which was statistically significant (Z = 5.93; P < 0.00001; I^2^ = 0%; Fig. 3B).

Seven studies investigated the effect of antibiotic treatment on cognitive performance on subjects with cognitive impairments whereas five studies investigated the effect of antibiotic on subjects with no prior cognitive impairments (Fig. 3C). Antibiotic treatment on subjects with existing cognitive impairment did not improve cognitive performance with a pooled effect estimate of − 0.11 (95% CI − 0.35, − 0.13; total number for control subjects = 485, antibiotic subjects = 468; Z = 0.87; P = 0.39). On the other hand, antibiotic treatment on subjects with no prior cognitive impairment worsen cognitive performance later in life with a pooled effect estimate of − 0.11 (95% CI − 0.14, − 0.07; total number for control subjects = 35,611, antibiotic subjects = 6831; Z = 5.93; P < 0.00001).

## Discussion

Our systematic review and meta-analysis is the first to analyse the effects of long-term or recurrent antibiotic use on cognitive performance. Our meta-analysis showed that long-term (≥ 15 days) or recurrent antibiotic treatment had a negative effect on cognition. In addition, our subgroup analysis of adult and pediatric patients revealed similar cognitive impairments, suggesting that long-term antibiotic use induced cognitive deficits irrespective of age. Our subgroup analysis revealed that when the studies were stratified according to the measurement of acute or chronic cognitive performance, long-term or recurrent antibiotic use only affected chronic cognitive performance. While this is interesting, and may suggest that the resulting cognitive deficit may take a while to manifest, it is important to point out that this subgroup analysis is not appropriately powered. Furthermore, the weighting is overwhelming towards the chronic subgroup. Nevertheless, our finding warrants further investigation in well-designed trials evaluating the time course of cognitive deficits following antibiotic treatments. Interestingly, all the pediatric studies evaluated cognitive performance several years after long-term or recurrent antibiotic use suggesting a chronic effect of antibiotic at an important age for development. Our meta-analysis included study populations that had prior cognitive deficits and those without. This can potentially confound the effects of antibiotics on cognitive performance. Nevertheless, the lack of heterogeneity in our meta-analysis supports the overall finding that there is a positive association of antibiotics use and cognitive impairments.

The clinical trials included in our meta-analysis investigated the cognitive enhancing effects of broad spectrum antibiotics doxycycline, minocycline, rifampin, d-cycloserine and rapamycin on patients with neurodegenerative diseases^22,24,26–28^. The dosages of antibiotics used in the included studies (Table 1) all fall within the recommended range for clinical use (National Institute for Health and Care Excellence UK guidelines). The recommended dosages for doxycycline, including minocycline, is 200 mg on day 1 and then 100 mg daily for most infections up to 500 mg twice a day for skin and soft tissue infections. Rifampin is used at 600 mg per dose for treatment of *Haemophilus influenzae* type b disease, meningococcal meningitis, leprosy and once daily for 6 months for the treatment of tuberculosis. d-cycloserine is also used to treat tuberculosis at 250 mg every 12 h for 2 weeks, up to 500 mg every 12 h. Sirolimus, another name for rapamycin, is indicated for 2 mg daily for 8 weeks for the prophylaxis of organ rejection in kidney allograft recipients.

Of all the clinical studies, only Loeb et al*.*^25^ concluded that doxycycline and rifampin treatment improved cognitive outcomes in patients with mild to moderate AD. However, a larger study testing the same antibiotics found no beneficial effects of doxycycline and rifampin on cognitive function in AD patients^26^. In fact, the trial found significant deterioration in cognition in doxycycline- and rifampin-treated groups compared to the placebo group. The authors suggested that the deterioration in the antibiotic-treated group was due to a loss of effect of cholinesterase inhibitors, a drug that 94% of the tested population was taking, and a known cognitive enhancer^41^. Rifampin is a potent inducer of CYP3A4 and would therefore increase the rate of elimination of cholinesterase inhibitors^42^. Kraig et al.^34^ tested the effects of 8 weeks of 1 mg rapamycin per day on cognition in 25 adults aged 70–95. The authors found no clinically significant effect of rapamycin and concluded that the trial duration was too short to observe cognitive improvements. Nevertheless, the dosage of rapamycin was found to be safely tolerated with no change in participants blood glucose concentration, insulin secretion, and insulin sensitivity. Similarly, the clinical trial testing d-cycloserine^35^, an agonist of the glutamatergic *N*-methyl-d-aspartate (NMDA) receptor, found no significant improvement in cognitive function over placebo at any point of the trial. The authors pointed out that participants of the trial received cognitive behavioural therapy (CBT) prior to each administration of d-cycloserine or placebo and hence, they were not able to measure further cognitive improvement in addition to that as a result of CBT. The other four randomised clinical trials (RCT) included in this meta-analysis investigated the effects of minocycline on cognitive improvement with none showing any beneficial effects of minocycline^24,27,28,39^. These studies concluded that the doses or duration of treatment with minocycline may have not been high enough or long enough to cause clinically relevant improvements in cognition. Howard et al*.*^24^ studied the effects of a 400 mg dose of minocycline administered for 2 years and found that it was poorly tolerated by the test group with a high dropout rate. Since the trial was designed to detect minimal clinically important difference between minocycline and placebo groups, the authors concluded that minocycline had no clinical benefit for AD.

All the observational cohort and case-controlled studies included in the meta-analysis investigated the chronic effects of long-term or recurrent antibiotic use on cognitive outcomes. In mice, long-term and recurrent treatment with antibiotics resulted in hippocampal-dependent cognitive deficits as well as altered gut microbial profiles^20^. In humans, chronic use of antibiotic is associated with early childhood obesity^43^ as well as increased risk of several types of cancer^44^. Both studies suggested a key role in antibiotic effects on the intestinal microbiome. There is a strong link between the gut dysbiosis and obesity^3^. A human study with obese and non-obese individuals found lower bacterial diversity in the gut of the obese group^45^. In rodent studies, there are reports of decreased Firmicutes and Bacteroidetes, major phyla of gut microbiota involved in lipid and bile acid metabolism, in obese mice^46,47^. Other studies found a regulatory role in the metabolites released by the gut microbiome on the release of metabolic hormones from enteroendocrine cells (for review see Martin et al.^48^). There is a strong link between obesity, gut microbiome dysbiosis and cognitive function in humans and in rodents (for review see Leigh and Morris^49^). Various mechanisms of action link the gut microbiota to brain function. Studies in antibiotic-treated mice found altered circulating metabolites, likely due to a depletion of the short-chain fatty acids (SCFAs) as a product of microbial fermentation in the gut^20^. These metabolites have been proposed to act as messengers in the communication between the gut microbiota and the brain. Interestingly, Park et al.^16^ found an increase in SCFAs in the AD patient’s brain after FMT suggesting that the restoration of dementia-related functions may be associated with an increase in SCFA levels.

Antibiotic treatment can significantly change the microbiome composition in children and adults. Antibiotic exposure in children has also been associated with several diseases risks including obesity, asthma, allergies and autoimmune disease^50–53^. Antibiotic exposure in the first 2 years of life particularly lead to a change in α-diversity^52^. There is evidence that microbiota diversity re-establishes after antibiotic treatment in infants as the gut microbiome matures^51^. However, long-term metabolic effects such as increased risk of high-fat diet induced obesity persisted after antibiotic cessation. Microbiome α-diversity in infants has been linked to lower cognitive ability at 2 years of age^54^. There are many factors that can affect cognitive progress at this important stage of development. Disturbance in gut microbiota diversity has been linked to decreased brain-derived neurotrophic factor (BDNF)^20^. BDNF has important functions in synaptic plasticity and neurogenesis, is an important neurotrophic factor for neuronal growth and development and its expression is intricately orchestrated at stages of brain development^55^. The levels of BDNF is also reduced in neurodegenerative diseases such as AD^56^. It is then unsurprising that our analysis found that antibiotic treatment impaired cognitive performance irrespective of the age of treatment and the age of cognitive assessment.

In addition to gut microbiome dysbiosis, a lot of different factors in these studies can affect cognitive abilities. In RCTs, participants were randomly assigned to antibiotic or control groups. However, even for the two largest trials included in this study, Howard et al.^24^ and Molloy et al.^26^, treatment allocation did not take into account socioeconomical status, educational attainment or any lifestyle factor such as diet that can affect cognitive assessment outcomes. In the observational studies included, one study (Mehta et al.^18^) adjusted for body mass index, regular use of antidepressants or depression symptoms, smoking status, regular use of multivitamins, high blood pressure, high cholesterol, type 2 diabetes, emphysema, history of stroke, history of myocardial infarction, regular use of aspirin or nonsteroidal anti-inflammatory drugs, physical activity, and dietary scores. In the studies with paediatric participants, Liu et al.^23^ compared the proportion of cognitive impairment by sociodemographic factors (e.g., gender, age, educational qualification, ethnicity, and income level), and clinical conditions and medical histories (e.g., Apoe4, smoking history, drinking history, BMI, history of hypertension, and diabetes) and Slykerman et al.^22^ adjusted for potential confounders including treatment group assignment, mode of delivery, breastfeeding and income. However, it remains that participants taking long-term or recurrent antibiotics may be at a poorer state of health in general, contributing to increased cognitive decline compared to their control counterparts.

There are a number of limitations with our systematic review and meta-analysis. The low number of studies precluded analyses for publication bias. The total number studied in the control group was 35,611 and 6831 in the antibiotic group. However, given the fact that an improvement or any deterioration in cognitive outcome after antibiotics would be a significant finding, we do not believe that there are any studies with unpublished results. However, some authors in the included studies had had funding from or serve as advisories for pharmaceuticals. Although none of the studies were funded by these companies, this still raises the possibility that there may have been publication bias. Two large studies in the meta-analysis carried a significant weight to the analysis whereas the remaining ten studies carried little weight; this also serves as a weakness to our analyses. To ensure that the effect of antibiotics on cognition is not based on a particular study, we carried out sensitivity analysis by removing individual studies from the meta-analysis. Our finding concludes that removing the heaviest weighted study showed a smaller but nevertheless statistically significant overall effect of antibiotic on cognitive performance. Another limitation of this study is based on the difference in cognitive test scores between control and antibiotic group, however, the type of cognitive test used in each study is different. Tests used include the Standardized mini mental state examination (sMMSE), Standardized Alzheimer’s Disease Assessment Scale-Cognitive Subscale (SADAS-cog), Fluid Intelligence (FI), Global Cognition CogState, Development Index of the Merrill-Palmer Revised Scale (DI), Neuropsychological Test Composite z score (NPZ-8) and Wechsler Abbreviated Scale of Intelligence (WISC). The variety of tests used is due to the underlying cognitive state of the test population. Participants with AD were tested with the sMMSE or SADAS-cog. The sMMSE is designed to test cognitive performance of older adults. It provides a global score of cognitive ability that correlates with daily function by assessing orientation to time and place, registration, concentration, short-term recall, naming familiar items, repeating a common expression, and the ability to read and follow written instructions, write a sentence, construct a diagram, and follow a three-step verbal command^57^. SADAS-cog provides a measure of change in the cognitive and behavioral functions known to be impaired by Alzheimer’s disease and is often used to test the effect of an intervention^58^. SADAS-cog measures verbal learning/memory, expressive language, receptive language, orientation to time and place, ideational praxis, and constructional praxis (figure copying). It also measures language ability and ability to follow test instructions^59^. Studies involving children used different tests to assess cognitive development. FI assesses children’s ability to reason and their ability to solve new problems and is related to working memory, attention and executive functions^60^. The DI-MPR evaluates general cognitive, social-emotional, self-help, receptive language, and fine and gross motor development in infants and children while providing supplemental scores for memory, speed of cognition, and visual-motor ability^61^. The NPZ-8 test assessed grooved pegboard performance, trail making, choice and sequential reaction, timed gait and symbol digit. Although the NPZ-8 comprises a heavy component of psychomotor speed testing, it also tests attention, executive functioning and memory^62^. The WISC tests general intellectual ability and assesses verbal comprehension, perceptual reasoning, working memory and processing speed^63^. Although each cognitive test assesses slightly different cognitive components, they all comprise of a number of cognitive functions to measure a general cognitive score. This is reflected in our meta-analysis showing that despite the heterogeneity in the tests used, antibiotics had a robust negative effect on cognition.

This systematic review highlighted the effects of antibiotics on cognition later in life. This calls for future well-designed and well-powered studies to investigate the impact of antibiotics on cognitive performance. This systematic review, the included studies, as well as future well conducted clinical studies are also important for determining clinical guidelines for safe and responsible use of antibiotics.

## Materials and methods

This systematic review and meta-analysis followed the Preferred Reporting Items for Systematic Reviews and Meta-analyses guidelines. No experiments on humans and/or human tissue samples were used. No Ethical or Institutional Review Board approval was required for the study design.

### Study design

This systematic review is reported according to the Preferred Reporting Items for Systematic reviews and Meta Analyses (PRISMA) guidelines^64^. We also followed the approaches outlined in the Cochrane Handbook for Systematic Reviews of Intervention^65^. We have established a priori protocol using the PRISMA-P (Preferred Reporting Items for Systematic review and Meta-Analysis Protocols) 2015 guidelines. This protocol has been registered on PROSPERO (protocol ID CRD42022358711) prior to study screening. With the exception of subgroup analyses and the definition of long-term antibiotic use, there were no protocol deviations in the conduct of this systematic review and meta-analysis.

### Eligibility criteria

#### Types of studies

We included all randomised and non-randomised controlled clinical trials, retrospective, and prospective observational studies (cohort studies, case-controlled studies, case-series) for our systematic review. We excluded case-reports, in vitro investigations or animal studies. We also restricted the language of the studies to English only and articles published until 07 Mar 2023.

#### Types of participants

We included studies with participants of any age. Studies with children under 2 years of age were included for any amount of antibiotics. For participants of any other ages, we included studies evaluating the effect of long-term or recurrent antibiotics. We define long-term antibiotic use as ≥ 7 days, and we define recurrent use of antibiotics as three or more separate prescriptions within a year.

#### Types of interventions and controls

We included any studies which evaluated the effect of any antibiotic administered via any route compared to no antibiotics or placebo. Any studies not comparing antibiotic use against the absence antibiotics or placebo were excluded.

#### Types of outcome measures

We included studies which recorded any type of cognitive assessment and assessment/risk of dementia (which include incidences of dementia) subsequent to antibiotic use. We excluded articles with no cognitive assessment after antibiotic treatment.

### Information sources

#### Study search

We searched for all English articles on PUBMED, EMBASE, ISI Web of Science, Scopus, and the Cochrane Central Register of Controlled Trials (CENTRAL) (The Cochrane Library) until 07 Mar 2023. In addition, manual searches through the bibliographies of publications that meet the eligible criteria for a full-text screening was carried out. We also searched clinicaltrials.gov for unpublished studies with results.

### Search strategy

Detailed search strategy for all electronic databases is included in the Supplementary Table 1.

### Study records

#### Data management

Records from electronic databases were downloaded into Endnote and Mendeley and scanned for duplicates. The article title, author names, journal, page numbers and DOIs were manually checked before duplicated records were removed. The titles and abstracts of electronic records identified from our search were uploaded to Rayyan.ai for title and abstract screening.

#### Selection process

Four authors (YY, HYT, WHC and ZQL) independently screened the titles and abstracts identified by the search strategy. After the initial screen, the full text of included articles was retrieved and screened according to the eligibility criteria defined above. Three senior authors (DBH, PHKT and OM) were consulted for conflict resolutions. Resolution of any conflicts involved a unanimous agreement from all study authors.

A flowchart according to the PRISMA guidelines was produced to outline the number of studies identified from each source, number of studies screened, retrieved and assessed for eligibility, and number of studies included and excluded from the systematic review and meta-analysis.

#### Data collection process

We extracted data from eligible studies using a modified data extraction form previously available from the Cochrane Library (available at https://epoc.cochrane.org/sites/epoc.cochrane.org/files/public/uploads/Resources-for-authors2017/good_practice_data_extraction_form.doc). Data extraction was performed by YY and HYT on all eligible studies. OM, DBH and PKHT were consulted for conflict resolutions with data extraction. We extracted the generic and the trade name of the antibiotic used, the type of control used, dosage, frequency and duration of treatment, patient characteristics (age when antibiotic treatment was administered and when cognitive tests were performed, gender, type of infections leading to the use of antibiotic if available), type of cognitive outcome measured, trial design, trial size, duration between antibiotic use and subsequent cognitive testing, type and source of financial support and publication status from trial reports. Where necessary, we used information from figures in the reports to approximate dispersion from means. Data was extracted from figures using a data extraction tool available at https://apps.automeris.io/wpd/^66,67^. A summary table of the type and dose of antibiotic used and how cognition was recorded was created.

### Outcomes and prioritization

Primary outcome measures include cognitive abilities after antibiotic use. Outcome measures were extracted separately in studies where two different antibiotics were evaluated on separate populations. The highest dose of antibiotic or the longest duration used were extracted for analysis if more than one dose or more than one period of treatment were evaluated.

All retrospective cohort studies included reported hazard ratios for dementia diagnosis after antibiotic use. One study reported odds ratio of cognitive impairment. The odds ratio was converted to SMD using the method described below. For all other studies, a cognitive score was obtained after one of these cognitive tests were performed: sMMSE, MCCB, SLUMS, fluid intelligence, CY-BOCS, CogState, SADAS-cog, Development Index of the MP-R Scale, neuropsychological z score, and Wechsler Abbreviated Scale of Intelligence (Table 1). Although each test assesses a different aspect of brain function, each of these test measures components of memory, attention, and executive function. The standardised mean difference (SMD) between control and antibiotic in each study was calculated to measure the difference between the two groups and compared between studies.

### Risk of bias assessment

We evaluated bias using the “Risk of Bias 2” (RoB2) tool from the Cochrane Foundation for randomised and non-randomised prospective clinical trials. The quality of RCT data for the articles included for the meta-analysis addressed the risk of bias in 5 main domains including risk of bias arising from the randomization process, risk of bias due to deviations from the intended interventions, risk of bias due to missing outcome data, risk of bias in measurement of the outcome and risk of bias in selection of the reported result. The study is judged to have a high risk of bias if there was a high risk of bias in one of the 5 domains. The risk of bias of cohort and case–control studies were graded using the Newcastle Ottawa Scale (NOS) based on the selection of participants, comparability of cohorts, and outcome assessment. A modified NOS which focuses on definition of case and control, comparability between case and control, ascertainment of exposure and response rate was used to assess risk of bias for case–control studies. A star is given to each assessment criteria on the NOS where 0–5 stars represent high risk of bias, 6–7 medium risk and 8–9 low risk of bias.

### Data synthesis and statistical analyses

Scores arising from different cognitive test batteries in individual studies were converted to a SMD using the following equation:

$$SMD=\frac{Difference\, in\, mean\, cognitive\, score}{SD\, of\, cognitive\, score}$$

We used baseline-adjusted scores in the evaluation of SMD. Studies which investigated cognitive outcomes after antibiotic use in populations without cognitive impairments had no baseline cognitive scores. It was therefore assumed that there was no difference in cognitive abilities between groups at baseline. For studies where higher tests scores indicate poorer cognitive performance, the SMD was multiplied by − 1. In studies reporting odds ratios (OR) for cognitive impairment, the SMD was obtained using the following formula:

$$SMD=\frac{{\text{ln}}(OR)}{\pi /\surd 3}$$

The standard deviation (SD) of the cognitive scores were calculated using reported 95% confidence intervals or standard error of means. The pooled effect estimate was a weighted SMD calculated using the inverse variance method under the assumption of random effects, with Review Manager 5.4^68^. Heterogeneity among studies was evaluated using the χ^2^ test, and a P-value of less than 0.05 was considered significant heterogeneity. We explored the extent of data heterogeneity with I^2^ statistics using the following boundaries: < 30% low heterogeneity; 30–60% moderate heterogeneity; > 60% substantial heterogeneity. Possible reasons for heterogeneity was explored using sensitivity analyses by removing individual studies from the analysis and evaluating the effect on the pooled estimate and heterogeneity.

The generic inverse variance was used to analyse the log hazard ratio and the standard error for outcomes from population studies that reported hazard ratios. A fixed effect model was used to plot the hazard ratios with 95% confidence intervals for these studies. To avoid over transformation of this data, hazard ratios were reported separately to SMD outcomes.

#### Subgroup analyses

We performed subgroup analysis for cognitive deficits according to participant age (pediatric and adult), participants with or without cognitive deficits at baseline, and studies which evaluated acute or chronic cognitive deficits.

### Supplementary Information

Supplementary Information 1.

Supplementary Information 2.

## Data Availability

The datasets generated during and/or analysed during the current study are available from the corresponding author on reasonable request.
