# Supplementary material for: A systematic review and meta-analysis of the effects of long-term antibiotic use on cognitive outcomes
Source: Sci Rep. 2024 Feb 18;14:4026. doi: 10.1038/s41598-024-54553-4 (PMC10874946; doi:10.1038/s41598-024-54553-4)
Supplement: Supplementary file 2 — Supplementary Information 2. [file 41598_2024_54553_MOESM2_ESM.pdf]

**SUPPLEMENTARY FIGURES LEGEND**

**Supplementary Table 1**

| **Source** | | **Search strategy** |
|---|---|---|
| 1. MEDLINE | 1 | (Antibacterial Agents OR Antibacterial Agent OR Anti-Bacterial Agent OR Anti Bacterial Agent* OR Anti-Bacterial Compound OR Anti Bacterial Compound* OR Bacteriocidal Agent* OR Bacteriocide* OR Anti-Mycobacterial Agents OR Anti-Mycobacterial Agent OR Antimycobacterial Agent* OR Antibiotic* OR Doxycycline OR Doxycycl* OR Vibramycin OR Atridox OR Deoxyoxytetracycline OR Periostat OR Vibravenos OR Benemycin OR Rifampicin OR Rifadin OR Rimactane OR Redactiv OR Xifaxan OR minocycl* OR Minomycin OR Rapamycin OR Rapamune OR Moxifloxacin OR Proflox OR Avelox OR Thalidomide OR Thalomid) OR (Antibacterial Agents OR Antibacterial Agent OR Anti-Bacterial Agent OR Anti Bacterial Agent* OR Anti-Bacterial Compound OR Anti Bacterial Compound* OR Bacteriocidal Agent* OR Bacteriocide* OR Anti-Mycobacterial Agents OR Anti-Mycobacterial Agent OR Antimycobacterial Agent* OR Antibiotic* OR Doxycycline OR Doxycycl* OR Vibramycin OR Atridox OR Deoxyoxytetracycline OR Periostat OR Vibravenos OR Benemycin OR Rifampicin OR Rifampin OR Rifadin OR Rimactane OR Redactiv OR Xifaxan OR minocycl* OR Minomycin OR Rapamycin OR Rapamune OR Moxifloxacin OR Proflox OR Avelox OR Thalidomide OR Thalomid) |
| | 2 | Dementia OR Amentia* OR Memory Disorder OR Cognitive Retention Disorder* OR Memory Loss* OR Memory Deficit* OR Cognitive Dysfunction* OR cognitive function OR Cognitive Impairment* OR Mild Cognitive Impairment OR Mild Neurocognitive Disorder* OR Cognitive Decline* OR Mental Deterioration* OR neurocognitive OR neurocog* OR cognt* OR alzheimer* OR MoCA OR MMSE |
| | 3 | woman OR women OR men OR man OR children OR paediatric OR pediatric OR pediatr* OR paediatr* OR geriatric OR geriatr* OR elderly OR elder* |
| | 4 | ((((((((((randomized controlled trial [pt]) OR (controlled clinical trial [pt])) ) OR (randomi* [tiab])) OR (placebo [tiab])) OR (drug therapy [all])) OR (randomly [tiab])) OR (trial [tiab])) OR (groups [tiab])) OR (case-control [all])) OR (participant* [tiab])) OR (outcome* [tiab]) |

| | | |
|---|---|---|
| | 5 | ((review [pt]) OR (review* [pt])) |
| | 6 | animals [mh] NOT humans [mh] |
| | 7 | #1 AND #2 AND #3 AND #4 NOT #5 NOT #6 |
| 2. EMBASE | 1 | 'antibacterial agents':ab,ti OR 'antibacterial agent':ab,ti OR 'anti-bacterial agents':ab,ti OR 'anti-bacterial agent':ab,ti OR 'anti-bacterial agent*':ab,ti OR 'antibacterial agent*':ab,ti OR 'anti-bacterial compound':ab,ti OR 'anti bacterial compound*':ab,ti OR 'bacteriocidal agent*':ab,ti OR 'bacteriocide*':ab,ti OR 'anti-mycobacterial agents':ab,ti OR 'anti-mycobacterial agent':ab,ti OR 'antimycobacterial agent*':ab,ti OR 'antibiotic*':ab,ti OR 'antibiotics':ab,ti OR 'antibiotic':ab,ti OR 'doxycycline':ab,ti OR 'doxycycl*':ab,ti OR 'vibramycin':ab,ti OR 'atridox':ab,ti OR 'deoxyoxytetracycline':ab,ti OR 'periostat':ab,ti OR 'vibravenos':ab,ti OR 'benemycin':ab,ti OR 'rifampicin':ab,ti OR 'rifadin':ab,ti OR 'rimactane':ab,ti OR 'xifaxan':ab,ti OR 'minocycl*':ab,ti OR 'minomycin':ab,ti OR 'rapamune':ab,ti OR 'moxifloxacin':ab,ti OR 'proflox':ab,ti OR 'avelox':ab,ti OR 'thalidomide':ab,ti |
| | 2 | 'dementia':ab,ti OR 'amentia*':ab,ti OR 'memory disorder':ab,ti OR 'cognitive retention disorder*':ab,ti OR 'memory loss*':ab,ti OR 'memory deficit*':ab,ti OR memory*:ab,ti OR 'cognitive dysfunction*':ab,ti OR 'cognitive function':ab,ti OR 'cognitive impairment*':ab,ti OR 'mild neurocognitive disorder*':ab,ti OR 'cognitive decline*':ab,ti OR 'mental deterioration*':ab,ti OR neurocognitive:ab,ti OR neurocog*:ab,ti OR cognit*:ab,ti OR alzheimer*:ab,ti OR moca:ab,ti OR mmse:ab,ti OR ((cognit*:ab,ti OR mental*) AND adj3 AND (declin*:ab,ti OR impair*:ab,ti OR los*:ab,ti OR deteriorat*:ab,ti)) OR forgetfullness:ab,ti OR 'memory complain':ab,ti OR 'mild cognitive impairment':ab,ti OR mci:ab,ti OR amci:ab,ti OR nmci:ab,ti OR 'age-associated memory impairment':ab,ti OR aami:ab,ti OR 'age consistent memory impairment':ab,ti OR acmi:ab,ti OR 'age related cognitive decline':ab,ti OR arcd:ab,ti OR 'cognitive impairment with no dementia':ab,ti OR cind:ab,ti |
| | 3 | 'woman':ab,ti OR 'women':ab,ti OR 'men':ab,ti OR 'man':ab,ti OR 'children':ab,ti OR 'paediatric':ab,ti OR 'pediatric':ab,ti OR 'pediatr*':ab,ti OR 'paediatr*':ab,ti OR 'geriatric':ab,ti OR 'geriatr*':ab,ti OR 'elderly':ab,ti OR 'elder*':ab,ti |

| | | |
|---|---|---|
| | 4 | 'randomized controlled trial':it OR 'controlled clinical trial':it OR randomi*:ab,ti OR placebo:ab,ti OR 'drug therapy':ab,ti OR randomly:ab,ti OR trial:ab,ti OR groups:ab,ti OR 'case control*':ab,ti OR participant*:ab,ti OR outcome*:ab,ti |
| | 5 | review*:it |
| | 6 | [animals]/lim NOT [humans]/lim |
| | 7 | #1 AND #2 AND #3 AND #4 NOT #5 NOT #6 AND [embase]/lim AND ([article]/lim OR [article in press]/lim OR [data papers]/lim OR [preprint]/lim) |
| 3. COCHRANE LIBRARY | 1 | "antibacterial agents" OR "antibacterial agent" OR "anti-bacterial agents" OR "anti-bacterial agent" OR "anti-bacterial agent*" OR "antibacterial agent*" OR "anti-bacterial compound" OR "anti bacterial compound*" OR "bacteriocidal agent*" OR "bacteriocide*" OR "anti-mycobacterial agents" OR "anti-mycobacterial agent" OR "antimycobacterial agent*" OR "antibiotic*" OR "antibiotics" OR "antibiotic" OR "doxycycline" OR "doxycycl*" OR "vibramycin" OR "atridox" OR "deoxyoxytetracycline" OR "periostat" OR "vibravenos" OR "benemycin" OR "rifampicin" OR "rifadin" OR "rimactane" OR "xifaxan" OR "minocycl*" OR "minomycin" OR "rapamune"OR "moxifloxacin" OR "proflox" OR "avelox" OR "thalidomide""anti bacterial agents" OR "antibacterial agent" OR "anti-bacterial agents" OR "anti-bacterial agent" OR "anti-bacterial agent*" OR "antibacterial agent*" OR "anti-bacterial compound" OR "anti bacterial compound*" OR "bacteriocidal agent*" OR "bacteriocide*" OR "anti-mycobacterial agents" OR "anti-mycobacterial agent" OR "antimycobacterial agent*" OR "antibiotic*" OR "antibiotics" OR "antibiotic" OR "doxycycline" OR "doxycycl*" OR "vibramycin" OR "atridox" OR "deoxyoxytetracycline" OR "periostat" OR "vibravenos" OR "benemycin" OR "rifampicin" OR "rifadin" OR "rimactane" OR "xifaxan" OR "minocycl*" OR "minomycin" OR "rapamune" OR "moxifloxacin" OR "proflox" OR "avelox" OR "thalidomide" |
| | 2 | dementia OR amentia* OR "memory disorder" OR "cognitive retention disorder*" OR "cognit* declin*" OR "cognit* impair*" OR "cognit* los*" OR "mental* declin*" OR "mental* impair*" OR "mental* los*" OR "memory loss*" OR "memory deficit*" OR memory* OR "cognitive dysfunction*" OR "cognitive function" OR "cognitive impairment*" OR "mild cognitive impairment" OR "mild neurocognitive disorder*" OR "cognitive decline*" OR "mental deterioration*" OR neurocognitive OR neurocog* OR cognit* OR Alzheimer* OR moca OR mmse OR forgetfullness OR "memory complain" OR "mild cognitive impairment" OR mci OR amci OR nmci OR "age- |

| | | |
|---|---|---|
| | | associated memory impairment" OR aami OR "age consistent memory impairment" OR acmi OR "age related cognitive decline" OR arcd OR "cognitive impairment with no dementia" OR cind |
| | 3 | woman OR women OR men OR man OR children OR paediatric OR pediatric OR pediatr* OR paediatr* OR geriatric OR geriatr* OR elderly OR elder* |
| | 4 | randomized AND controlled AND trial OR "controlled clinical trial" OR randomi* OR placebo OR "drug therapy" OR randomly OR trial OR groups OR "case-control" OR participant* OR outcome* |
| | 5 | animal* NOT human* |
| | 6 | #1 AND #2 AND #3 AND #4 NOT #6 |
| 4. Web of Science – all editions | 1 | ((((((((((((((((((((((TS=(Antibacterial Agent*)) OR TS=((antibacterial compound*))) OR TS=((bacteriocidal agent*))) OR TS=((bacteriocide*) )) OR TS=((antimycobacterial agent*) )) OR TS=(antibiotic*)) OR TS=(doxycycl* )) OR TS=(vibramycin)) OR TS=(atridox )) OR TS=(deoxyoxytetracycline )) OR TS=(periostat)) OR TS=(vibravenos )) OR TS=(benemycin )) OR TS=(rifampicin)) OR TS=(rifadin )) OR TS=(rimactane)) OR TS=(xifaxan)) OR TS=(minocycl* )) OR TS=(minomycin)) OR TS=(rapamune)) OR TS=(moxifloxacin)) OR TS=(proflox)) OR TS=(avelox)) OR TS=(thalidomide) |
| | 2 | (((((((((((((((((((((((((((((((((((((TS=(dement*)) OR TS=(amentia*)) OR TS=(memory disorder*)) OR TS=(cognitive retention disorder*)) OR TS=(memory los*)) OR TS=(memory deficit*)) OR TS=(cognitive dysfunction*)) OR TS=(cognitive function*)) OR TS=(cognitive impairment*)) OR TS=(mild cognitive impairment*)) OR TS=(neurocognitive disorder*)) OR TS=(cognitive decline*)) OR TS=(mental deterioration*)) OR TS=(neurocog*)) OR TS=(cognit*)) OR TS=(alzheimer*)) OR TS=(MOCA*)) OR TS=(MMSE*)) OR TS=(memory*)) OR TS=(cognit* declin*)) OR TS=(cognit* impair*)) OR TS=(cognit* los*)) OR TS=(mental* declin*)) OR TS=(mental* los*)) OR TS=(mild neurocognitive disorder*)) OR TS=(forgetfullness)) OR TS=(memory complain)) OR TS=(mild cognitive impairment)) OR TS=(mci )) OR TS=(amci )) OR TS=(nmci)) OR TS=(age-associated memory impairment)) OR TS=(aami)) OR TS=(age consistent memory impairment)) OR TS=(acmi)) OR TS=(age related cognitive decline)) OR TS=(arcd)) OR TS=(cognitive impairment with no dementia)) OR TS=(cind) |

| | |
|---|---|
| | 3 (((((TS=(wom?n)) OR TS=(m?n)) OR TS=(children)) OR TS=(p*diatric)) OR TS=(p*diatry)) OR TS=(geriatr*)) OR TS=(elder*)<br>4 ((((((((TS=(outcome*)) OR TS=(participant*)) OR TS=(case-control*)) OR TS=(group*)) OR TS=(trial*)) OR TS=(random*)) OR TS=(drug therap* )) OR TS=(placebo)) OR TS=(controlled clinical trial*)) OR TS=(randomi?ed controlled trial*)<br>5 (ALL=(animal*)) NOT ALL=(Human*)<br>6 DT=(reivew)<br>7 #1 AND #2 AND #3 AND #4 NOT #5 NOT #6 |
| 5. Scopus | ( TITLE-ABS-KEY ( "antibacterial agents" OR "antibacterial agent" OR "anti-bacterial agents" OR "anti-bacterial agent" OR "anti-bacterial agent*" OR "antibacterial agent*" OR "anti-bacterial compound" OR "anti bacterial compound*" OR "bacteriocidal agent*" OR "bacteriocide*" OR "anti-mycobacterial agents" OR "anti-mycobacterial agent" OR "antimycobacterial agent*" OR "antibiotic*" OR "antibiotics" OR "antibiotic" OR "doxycycline" OR "doxycycl*" OR "vibramycin" OR "atridox" OR "deoxyoxytetracycline" OR "periostat" OR "vibravenos" OR "benemycin" OR "rifampicin" OR "rifadin" OR "rimactane" OR "xifaxan" OR "minocycl*" OR "minomycin" OR "rapamune" OR "moxifloxacin" OR "proflox" OR "avelox" OR "thalidomide" ) ) AND ( TITLE-ABS-KEY ( dementia OR amentia* OR "memory disorder" OR "cognitive retention disorder*" OR "cognit* declin*" OR "cognit* impair*" OR "cognit* los*" OR "mental* declin*" OR "mental* impair*" OR "mental* los*" OR "memory loss*" OR "memory deficit*" OR memory* OR "cognitive dysfunction*" OR "cognitive function" OR "cognitive impairment*" OR "mild cognitive impairment" OR "mild neurocognitive disorder*" OR "cognitive decline*" OR "mental deterioration*" OR neurocognitive OR neurocog* OR cognit* OR alzheimer* OR moca OR mmse OR forgetfulness OR "memory complain" OR "mild cognitive impairment" OR mci OR amci OR nmci OR "age-associated memory impairment" OR aami OR "age consistent memory impairment" OR acmi OR "age related cognitive decline" OR arcd OR "cognitive impairment with no dementia" OR cind ) ) AND ( TITLE-ABS-KEY ( woman OR women OR men OR man OR children OR paediatric OR pediatric OR pediatr* OR paediatr* OR geriatric OR geriatr* OR elderly OR elder* ) ) AND ( TITLE-ABS-KEY ( randomized AND controlled AND trial OR "controlled clinical trial" OR randomi* OR placebo OR "drug therapy" OR randomly OR trial OR groups OR "case-control" OR participant* OR outcome* ) ) AND NOT ( TITLE-ABS-KEY ( animal* AND NOT human* ) ) AND NOT ( DOCTYPE ( re ) ) |

| | |
|---|---|
| 6. Clinicaltrials.gov (www.clinicaltrials.gov) | dementia AND antibiotic |

**Supplementary Table 1.** Detailed search strategy for all electronic databases.

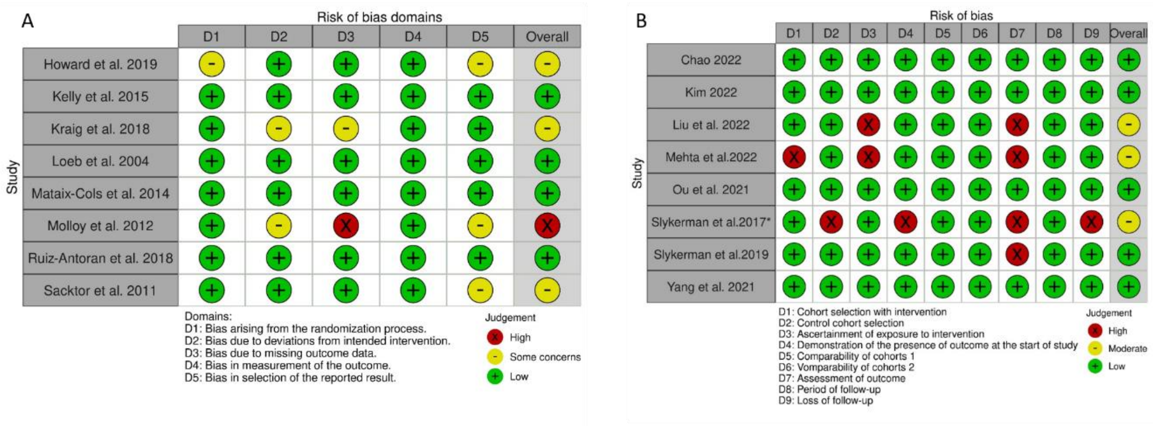


**Supplementary Figure 1.** Risk of bias assessments for included studies

**A)** Risk of bias analysis for 8 randomised controlled trials was determined using the "Risk of Bias 2" (RoB2) tool from the Cochrane Foundation for randomised and non-randomised prospective clinical trials. One study, Molloy et al. 2012, has a high risk of bias due to missing outcome data in the study. In addition, there are some concerns regarding selection of reported results Sacktor et al. 2011 and Howard et al. 2020, and deviations from intended intervention and bias due to missing outcome data in Kraig et al. 2018. **B)** The risk of bias of cohort and case-control studies were graded using a mondified Newcastle Ottawa Scale (NOS) based on the selection of participants, comparability of cohorts, and outcome assessment. A star is given to each assessment criteria on the NOS where 0-5 stars represent high risk of bias, 6-7 medium risk and 8-9 low risk of bias. 5 out of 8 studies had low risk of bias; 3 had a low to moderate risk of bias.

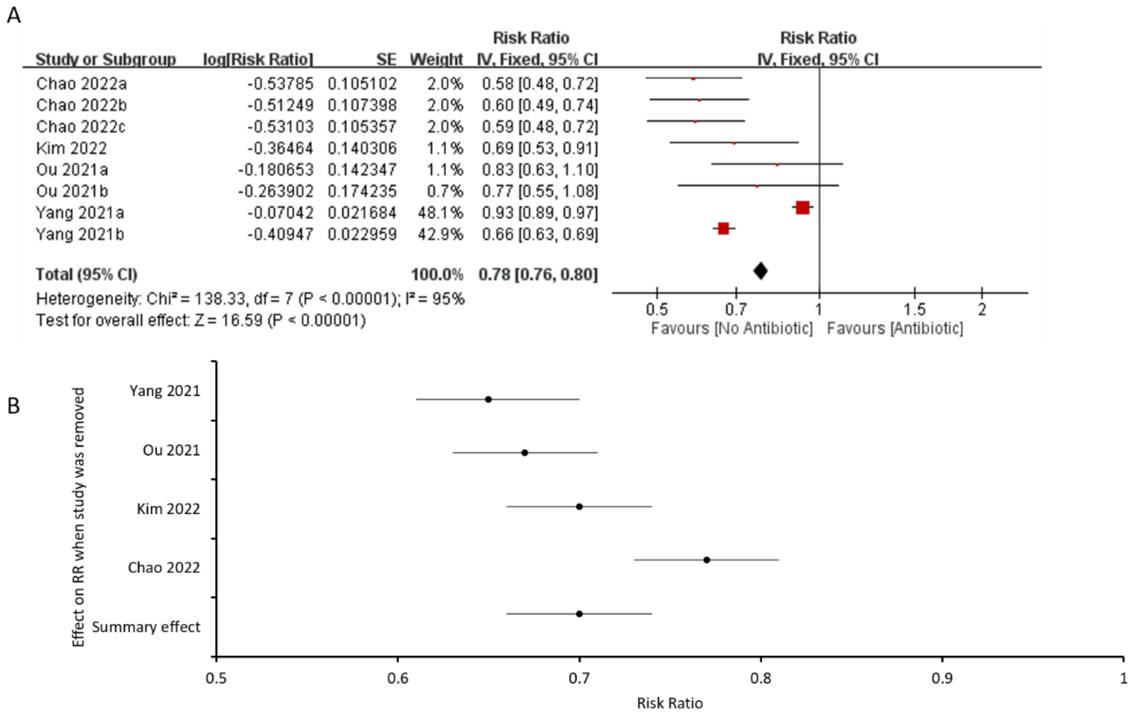


**Supplementary Figure 2.** The effects of antibiotics on cognition for studies which reported hazard ratios for the likelihood of developing dementia after antibiotic treatment.

**A)** The log risk ratio for the four retrospective cohort studies were analysed using the generic inverse-variance method and the pooled effect estimate was 0.78 [95% CI 0.76, 0.80]. A Z-test showed that antibiotic use was again associated with worse cognitive outcomes (Z = 16.59; P<0.00001; $\chi^2$ = 138.33, P < 0.00001; $I^2$ = 95%). This means that 95% of the variability in the effects observed is due to heterogeneity and not chance. **B)** Sensitivity analysis did not find a great deal of difference when studies were removed sequentially (risk ratios from 0.65 – 0.77).
